# Analysis and mitigation of equipment-induced shortcuts in AI models for laparoscopic cholecystectomy

**DOI:** 10.1371/journal.pdig.0001421

**Published:** 2026-10-08

**Authors:** Sergey Protserov, Anastasiia Repalo, Pouria Mashouri, Jaryd Hunter, Caterina Masino, Amin Madani, Michael Brudno

**Affiliations:** 1 Department of Computer Science, University of Toronto, Toronto, Ontario, Canada; 2 Vector Institute for Artificial Intelligence, Toronto, Ontario, Canada; 3 University Health Network, Toronto, Ontario, Canada; Aarhus University Hospital, DENMARK

## Abstract

Machine learning models have seen a lot of success in medical image segmentation domain. However, one of the challenges that they face are confounders or shortcuts: spurious correlations or biases in the training data that affect the resulting models. One example of such confounders for surgical machine learning is the setup of surgical equipment, including tools and lighting. Using the task of identification of safe and dangerous zones of dissection in laparoscopic cholecystectomy images and videos as a use-case, we inspect two equipment-induced biases: the location of surgical tools in the field of view and the direction of lighting, both of which are tightly related to the region of interest. We propose methods for evaluating the severity of these biases based on measurements of model consistency under real or simulated exposure to these shortcuts, and augmentation-based methods for mitigating them. We show that our tool bias mitigations based on pasting tool images in random locations improve the models’ prediction consistency under tool movements by 9 percentage points in the most inconsistent cases, and by 4 percentage points on average. Our lighting bias mitigations based on simulated lighting adjustments help reduce fraction of pixels originally predicted as belonging to the dangerous zone that may flip to safe under light changes from 5% to 1.5%, without compromising segmentation quality.

## Author summary

Artificial intelligence is increasingly used to support surgeons, for example by highlighting which tissue is safe to cut and which is dangerous during gallbladder removal (laparoscopic cholecystectomy), one of the most common abdominal operations. These systems can, however, learn the wrong lesson. We found that AI models for this task come to rely on where the surgeon places the instruments and where the camera light is pointed, rather than on the anatomy itself. Experienced surgeons tend to position their tools and lighting in predictable places, so the model takes a shortcut. The danger is that a less experienced surgeon, who handles equipment differently, could receive misleading guidance. This flaw is hard to detect because the pattern runs through all of the data, so ordinary testing does not reveal it. We developed a way to measure how strongly a model leans on these shortcuts, and a simple training method that breaks them by inserting instruments and extra lighting at random positions. Our approach makes predictions substantially more consistent without sacrificing accuracy, a step toward surgical AI that offers trustworthy guidance to surgeons at every level of experience.

## Introduction

Surgical complications represent a substantial burden on global healthcare systems, contributing significantly to patient morbidity, mortality, and economic costs. Research indicates [1,2] that a significant portion of surgical adverse events is caused by errors in perception and decision-making among surgeons.

The integration of artificial intelligence and machine learning technologies into surgical practice offers a promising approach to addressing these challenges by providing real-time decision support to surgical teams. Advanced deep learning algorithms have demonstrated [3] remarkable capabilities in surgical computer vision tasks, achieving performance comparable to that of expert surgeons for the tasks of anatomical structure identification.

In recent years, deep learning machine models based on convolutional [4] and transformer [5] neural networks have seen much success in the field of medical image segmentation, as evidenced by multiple systematic reviews [6–13]. However, clinical utility of such models is not necessarily directly reflected by segmentation evaluation metrics, such as Dice score, precision and recall. Deployment of these models to clinical environments and their real-world usefulness are hindered by confounders present in the input images [14], allowing the model to demonstrate high performance on held-out test data that does not generalize to other settings. Some examples of such confounders are information that allows the model to distinguish between different hospital systems or departments [15] which have different prevalence of outcome of interest, or location of the object in the field of view being correlated with the segmentation label [16].

In this paper we consider the problem of identifying safe and dangerous zones of dissection during laparoscopic cholecystectomy (LC). While prior work [17,18] has shown that computer vision techniques can have broad applicability to this problem, we have identified two medical equipment-related confounders that are present in training and testing datasets, and affect models developed using this data. Specifically, medical tool location in the field of view and the direction of lighting are both highly correlated with safe and dangerous zone locations, due to how this surgery is performed. The tool tips of dissection instruments (scalpels, cauterizers) are most often found in the safe zone, as this is exactly where the surgeons perform dissections. Similarly, lighting is typically directed to the same zone of interest. Direction of lighting may be further correlated with the tool location, potentially complicating the relationship between tool, lighting and zones of interest.

In this work we propose methods for evaluating the extent to which a given model is affected by these biases. For tool bias, we use both manually and automatically obtained tool segmentations to find pairs of frames that are similar except for tool locations, and assess model prediction consistencies between frames in such pairs. For lighting bias, we assess how model predictions change when different simulated lighting adjustments are applied to the input frames. We therefore show that the models trained on biased datasets are indeed confounded by tool and lighting locations. We further propose augmentation-based strategies for mitigation of these issues, based on pasting in images of surgical instruments in random positions and random local lighting adjustments to break the correlations with safe/dangerous zone location. We show that the best tool bias mitigation result is achieved when the tool pasting augmentation is applied twice per input image during training, and this helps improve the models’ consistency under tool movements by 9 percentage points in the most inconsistent cases, and by 4 percentage points on average, and that tool texture, and not only shape, plays a significant role in these augmentations. We also found that the worst case for lighting bias is when the lighting is directed to the regions inside the predicted safe zone, which is, importantly, the most surgically-relevant scenario, and in such cases our mitigations help reduce the fraction of pixels originally predicted as belonging to the dangerous zone that may be predicted as safe zone under different lighting conditions from 5% to 1.5%. Altogether, we demonstrate that our mitigation strategies indeed help train unbiased models, without compromising segmentation quality, and therefore improve the models’ clinical usefulness and reliability.

## Background work

Studies that consider the computer vision tasks related to laparoscopic surgery include EndoNet [19], where the authors develop a model for surgery phase recognition and tool detection in laparoscopic videos, DeepCVS [20], where the authors develop a model for highlighting hepatocystic anatomy and assessing whether the critical view of safety (CVS) has been established during laparoscopic cholecystectomy, and the study [17], where the authors develop models for highlighting hepatocystic anatomy and safe and dangerous zones of dissection during laparoscopic cholecystectomy. More recently Surgesture [21] focused on identifying the surgeon movements during laparoscopic cholecystectomy that can be used to quantify and summarize operative patterns of surgeons with different levels of expertise, while [18] further improved upon results from [17] and proceeded to deploy an optimized version of the model for safe/dangerous zone highlighting. Our work builds upon these publications, however focuses specifically on the biases introduced by medical equipment setup in the field of view. Finally, Zheng et al [22] describe a generative model-based system for improving the quality of laparoscopic videos by reducing image blur and removing smoke and camera fog. They demonstrate that the use of this system helps reduce the operation pause time and surgeon anxiety.

Another broad field of research relevant to this study is shortcut learning [14]. Confounders make models learn spurious correlations (shortcuts) in training data, instead of meaningful features. They are particularly insidious as they are likely to be also present in the test set built via a random split of the training data, leading to overconfidence in the performance of the model. One noteworthy example of such shortcuts is described in [15], where the authors found that convolutional neural networks trained for pneumonia screening from chest X-rays fail to generalize to external sites, while being able to predict which hospital system and even which hospital department a given image comes from. Analysis of activation maps showed that when predicting the source of a given image, the model “pays attention” to image corners, and laterality tokens specifically. The main finding of this paper is that when different hospital systems have different levels of pneumonia prevalence, the models can indirectly leverage this information to produce confounded predictions. The work [16] further studies shortcut learning in the context of medical image segmentation on two different datasets, demonstrating that presence of text and calipers in the fetal ultrasound images provides shortcuts for organ segmentation, and that centered location of skin lesions in the skin lesion segmentation dataset provides shortcuts that cause the models to under-perform if the lesion is not located at the center of the image at test time. That study shows that training on a cleaned-up version of the first dataset with text and calipers removed by inpainting, and applying random cropping augmentations to the second dataset, can help mitigate the respective biases. Our own augmentation-based bias evaluation and mitigation techniques are similar to these ideas proposed in [16]. The study [23] focuses on the shortcut in classifier models for skin cancer prediction which learn that in the ISIC dataset color calibration charts occur only in benign images, but not in malignant ones. The authors demonstrate the existence of this bias in trained models by artificially inserting these charts into malignant images, and inpainting them away in benign images. They further propose a de-biasing strategy based on inpainting the charts with benign skin pixels in biased images. We are not aware of any studies that focus specifically on shortcuts or confounders in surgical videos.

To reduce the impact of shortcuts, general-purpose shortcut learning mitigation strategies are available, such as Deep Feature Reweighting (DFR [24]), which amounts to re-training a model’s last layer on unbiased data, and MARG-CTRL [25] family of methods, which offers shortcut learning mitigation through various modifications of cross-entropy loss used during training.

## Methods

### Ethics statement

This research uses de-identified patient data, and has been approved by the University Health Network (UHN) Research Ethics Board, REB#20-5349.

### Data

In this research, we use the following datasets:

- The dataset previously described in [18] as Dataset 1 that consists of 2627 images from laparoscopic camera extracted from 289 LC videos across 136 different institutions from 37 countries and all continents, with pixel-level annotations of safe and dangerous zones of dissection available for every image. Safe zones correspond to the areas where a surgeon should perform dissections, while dangerous zones are locations where dissection is likely to result in an injury to the patient. We use this dataset and the problem of safe/dangerous zone segmentation for model bias analyses. The dataset was split into training, validation and testing subsets on a per-video basis, with 1843 images from 201 videos, 390 images from 43 videos and 394 images from 45 videos assigned to the corresponding sets. Out of the 2627 images, 2473 have surgical tools in them and 154 do not. An example of an annotated image from this dataset is shown in Fig 1.
- Laparoscopic cholecystectomy images from CholecT50 [26], Cholec80 [19], CholecSeg8k [27] and Endoscapes [28] datasets, used with surgical tool segmentation masks from CholecInstanceSeg [29] dataset to train a tool segmentation model. These image datasets overlap with each other and with Dataset 1 described in the first item, so we carefully analyzed the overlaps and selected only a subset of images to ensure no data leakage takes place. The resulting split is 20546 images from 33 videos for training, 6730 images from 11 videos for validation and 3275 images from 11 videos for testing.
- Two laparoscopic cholecystectomy videos from CholecTrack20 dataset [30] which are not present in any of the above mentioned datasets, used for scaling our tool bias mitigation evaluations to videos with model-provided (as opposed to manual) tool segmentations.

**Fig 1:**
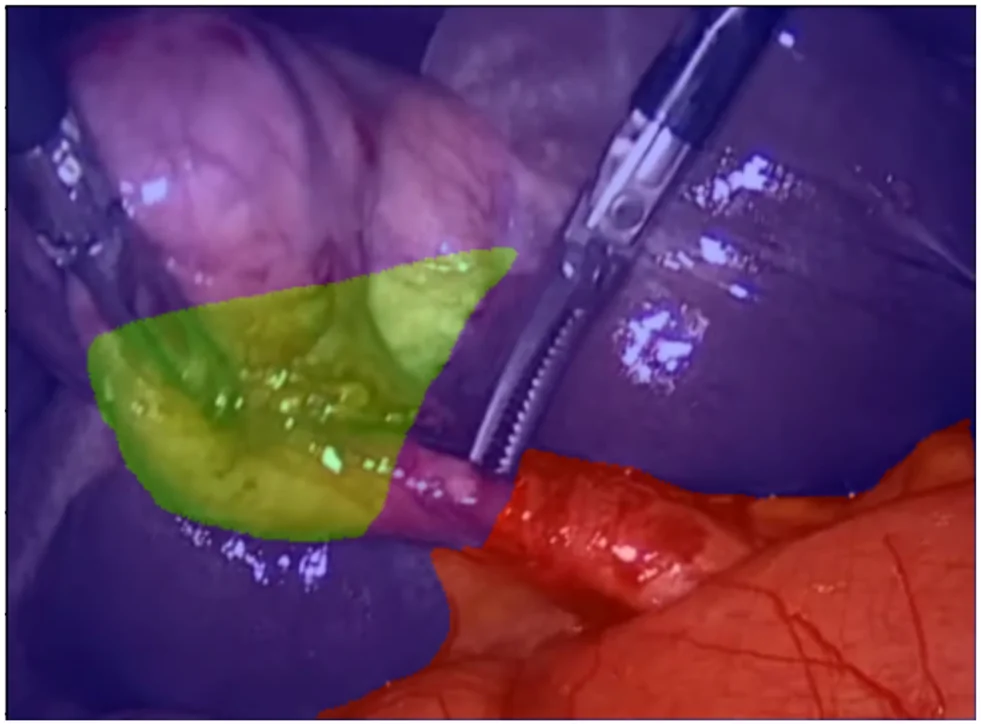
An example annotated image from the LC dataset. Red highlight shows dangerous zone, green – safe zone, blue – background.

For the purposes of model training and evaluation, all images and masks were downscaled to the height of 128 pixels, and cropped or padded to the width of 288 pixels. Our previous experiments with this data showed that this aspect ratio provides an excellent coverage of the true variety of frame dimensionalities, and that larger resolutions do not provide any improvement.

A dataset overview is given in S1 Table.

### Tool bias

#### Problem statement.

When conducting LC, the surgeons use a variety of surgical tools and instruments to perform a number of tasks, including dissecting, spreading, cutting or burning through tissues. Examples include scalpels, graspers and cauterization tools. If we consider the problem of identifying and highlighting safe and dangerous zones of dissection, it is clear that there is likely to be strong correlation between the positions of tools and the safe zone locations in any dataset for this problem (experienced surgeons are likely to only use tools in safe zones). Because of that, we hypothesize that models trained on such data will be biased by presence of the tools in the field of view, and may provide erroneous predictions depending on the actual location of the surgical tool. This would have the potential of being harmful if the tool is used by a less experienced surgeon, as the safe zone prediction will potentially “follow” the surgical tools.

#### General principles of evaluation.

Intuitively, a perfectly unbiased model would output the exact same predictions for two otherwise similar input frames that only differ in the positions of surgical tools. Our proposed tool bias evaluation methodology builds upon this idea, with the goal being to validate whether model’s predictions change due to the movement of the tool; the more they change, the more a model is affected by the presence of surgical instruments. Note that we can’t use safe/dangerous zone ground truth for tool bias assessment because that would require annotated frames that are temporally close enough to satisfy the “background is mostly static, only tools move” constraint. For each of the 289 surgical videos we have at most 10 annotated images that are spread uniformly along the video. Therefore, we use consistency of model predictions between similar frames as a proxy. The available ground truth is used to demonstrate that segmentation quality hasn’t dropped as a result of introducing the bias-mitigating augmentations in Section Segmentation performance. We implemented our tool bias evaluation methodology in two different variants, with manual or automated tool location tracking.

#### Manual evaluation.

We manually identified 23 short video fragments in the testing subset of videos, such that most of the movement in these fragments is attributed to surgical tools, and the background is mostly static. We then used Track Anything [31] program to interactively create frame-by-frame pixel-level annotations of surgical tools in these videos, while manually verifying every annotation. Then, for every such short video, we considered all frames with a step of 100 ms, and all possible pairs of these frames. We used Hausdorff distance between tool locations in the two frames, and mean squared distance between all non-tool pixels to identify frame pairs where the tools are relatively far apart, while the background is largely unchanged. For every video, we selected 50 frame pairs that best satisfied these criteria.

To evaluate a model for tool bias, we obtain the model’s predictions on all selected frames, and consider the predictions’ consistency between frames within every selected pair by measuring class average (average over the three segmentation classes: safe, dangerous and background) of Dice score between predictions obtained for both frames. We chose to use Dice score for this purpose because it is symmetric with respect to which image is considered as ground truth vs which is a predicted mask; this is important since the two frames within a pair are interchangeable.

#### Automated evaluation.

To demonstrate that manual tool tracking is not strictly necessary and our tool bias evaluation can be run at scale, we also performed the following steps to obtain more candidate frame pairs:

1. Trained a model for semantic segmentation of surgical tools on the CholecInstanceSeg [29] dataset
2. Used this model to obtain tool segmentations for the two videos from CholecTrack20 dataset [30]
3. Using a sliding window of 5 seconds over the relevant parts of these videos, considered pairs of frames within every window
4. Computed Hausdorff distances between tools, MSEs between non-tool pixels, and safe/dangerous zone segmentation consistencies of the baseline and augmented models for the frame pairs
5. Considered the frame pairs from the two videos together, binned them by MSE and plotted consistency improvement of the augmented model versus baseline depending on Hausdorff distances between tools in the frame pairs

#### Mitigations.

To mitigate the tool bias, we used 215 images of surgical tools extracted from the training subset of videos, including from frames that were never annotated for safe/dangerous zones and therefore never used in model training, and defined a tool pasting augmentation, which pastes tools in random positions in the image during training, properly adjusting their size to the size of the target image, and with random rotations. We hope that pasting surgical tools in random places in the input image will break the correlation between tool position and safe zone location, leading to models that are more robust in the presence of surgical instruments. The tools that were used for augmentation include scalpels, graspers and cauterizers, and cover the same tool groups that are present in the validation and testing data subsets. These extracted tool images reflect the diversity of the collected surgical video samples, and by only using the training subset of videos for tool extraction we guarantee that there is no data leakage into the testing subset and procedures. Some examples of raw tool images as extracted from the frames are given in S1 Fig, and examples of tool-augmented images are presented in Fig 2B-C.

**Fig 2:**
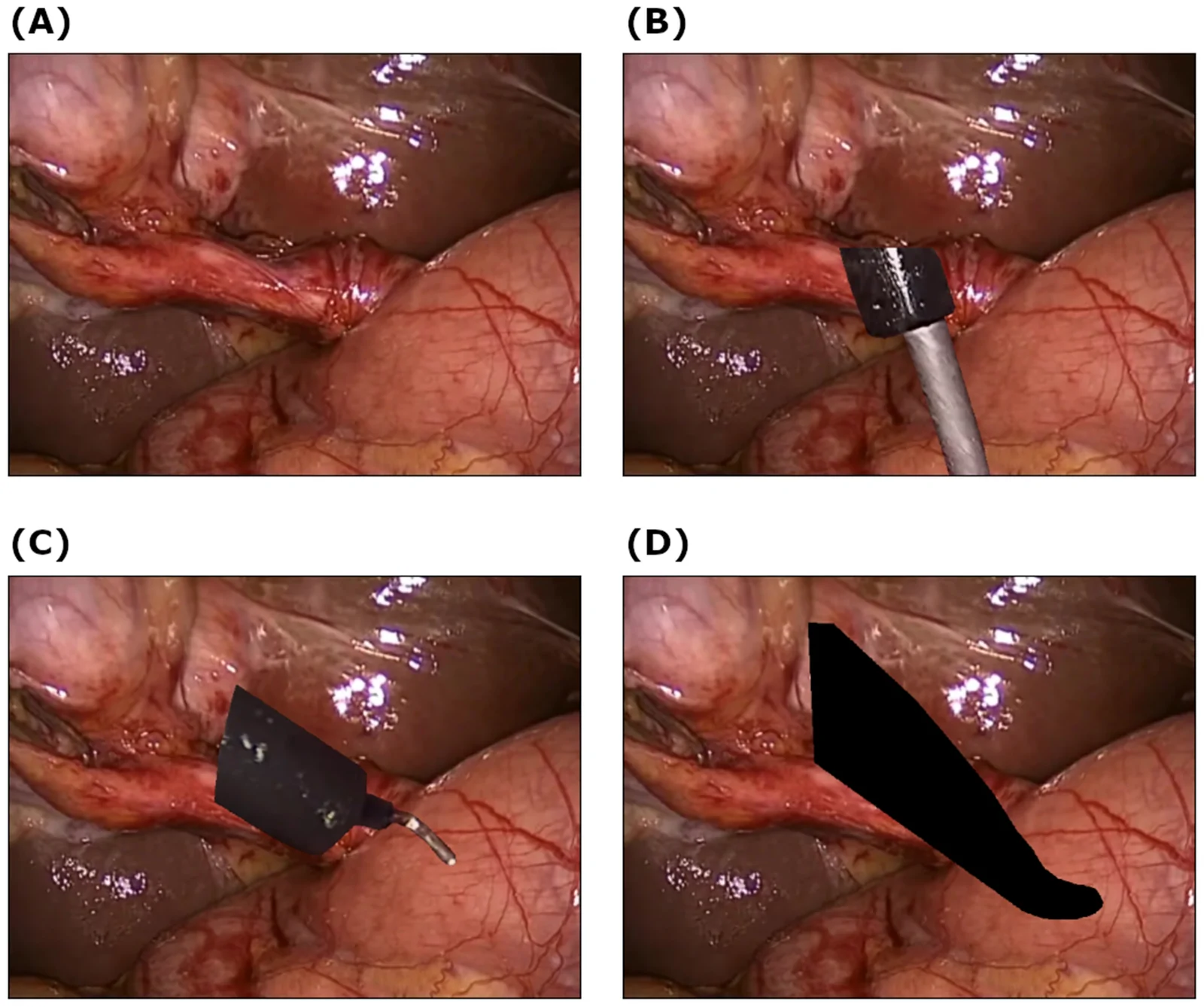
Examples of images augmented with our tool pasting augmentation. A: Original image, without tool augmentation. B: Augmented image 1. C: Augmented image 2. D: Augmented image 3. Here pasted tool is filled with black color to validate whether the texture of the tool is significant.

To simulate presence of multiple tools in the field of view and make our augmentation stronger, we also tried applying the tool pasting augmentation several times to the same image, with 1–3 tools being pasted into every image, depending on a model training hyperparameter.

It is important to note that tool pasting augmentation also has an implicit effect of covering large sections of the original input, which may serve as a strong regularization technique in itself. To study the effect obscuring parts of the image may have on the model, we also consider the augmentation mode where the pasted tool is filled completely with black color (Fig 2D). On average, applying tool pasting augmentation once obstructs 6% of the input image, and applying it twice obstructs 11.5% of the image.

### Lighting bias

#### Problem statement.

During LC, the light mounted on the laparoscopic camera is typically directed to the region of interest, where the dissection is being performed. Therefore, there is likely to be a correlation between direction of lighting and safe zone locations. This bias is also likely to be learned by the model, and may impact the performance of a system in a real-world deployment, especially when used by less experienced surgeons.

#### Evaluation.

To evaluate to what extent a given model suffers from lighting bias with a given input image, we first obtain the model’s prediction on this image, and then simulate additional lighting in different parts of the image. To study the effect directing lighting to different locations might have on predictions, we simulate lighting centered at different points in the predicted safe zone only, in the predicted dangerous zone only, or anywhere in the image. For every input image, for every lighting simulation mode, we created 50 transformed images. We then assess by how much predictions on the images with simulated lighting differ from the prediction on the original image. The intuition is that a model that is robust to lighting changes should have high consistency. We conduct this experiment on all images in the test subset, and aggregate the results across the images.

#### Mitigations.

Similarly to the idea of our tool pasting augmentation described in Section Mitigations, we implement a lighting augmentation that locally increases brightness of a random region within the image. The input image is converted from RGB to LAB color space, and a Gaussian blob is added to the L-channel with a center at a random position. The image is then converted back to RGB. For the purposes of evaluation, the intensity of the brightest point (center) of the Gaussian lighting blob is selected randomly between 20 and 50 L-channel points (which range from 0 to 100), for the purposes of augmentation it is fixed at 50 L-channel points. Some examples of images augmented in this manner are presented in Fig 3.

**Fig 3:**
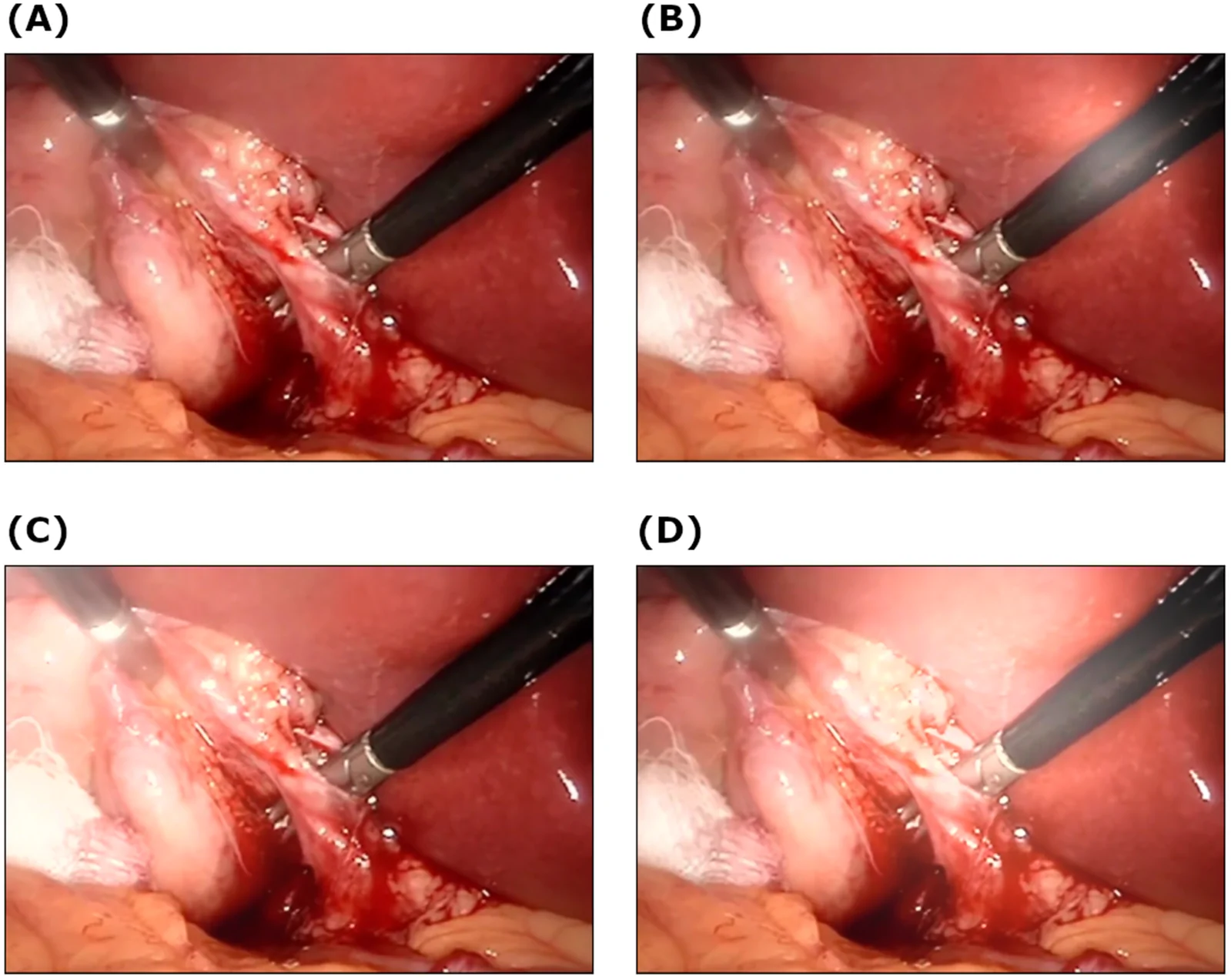
Examples of images augmented with our lighting augmentation. A: Original image, without lighting augmentation. B: Augmented image 1. C: Augmented image 2, D: Augmented image 3.

## Results

### Baseline setup

We used SegFormer [32] architecture, and followed the standard machine learning practice of training multiple models with different hyperparameter sets to identify the best model with respect to value of the utilized loss function, cross-entropy, on the validation set. The best model selected in this manner is then used for evaluation on the test set. In our earlier work [18] we showed that SegFormer performs on par with U-Net [33], another well-established semantic segmentation architecture. We resize every image to a height of 128 pixels while preserving the aspect ratio. Our previous work with this dataset showed that this resolution is sufficient. The brightest 100x100 square within the image is selected, and the entire image is standardized so that this square has zero per-channel mean and unit per-channel standard deviation. This strategy is used instead of “regular” standardization, because some inputs have black borders (see Fig 4A) which would distort computations of mean and standard deviation if they were performed over the entire image.

**Fig 4:**
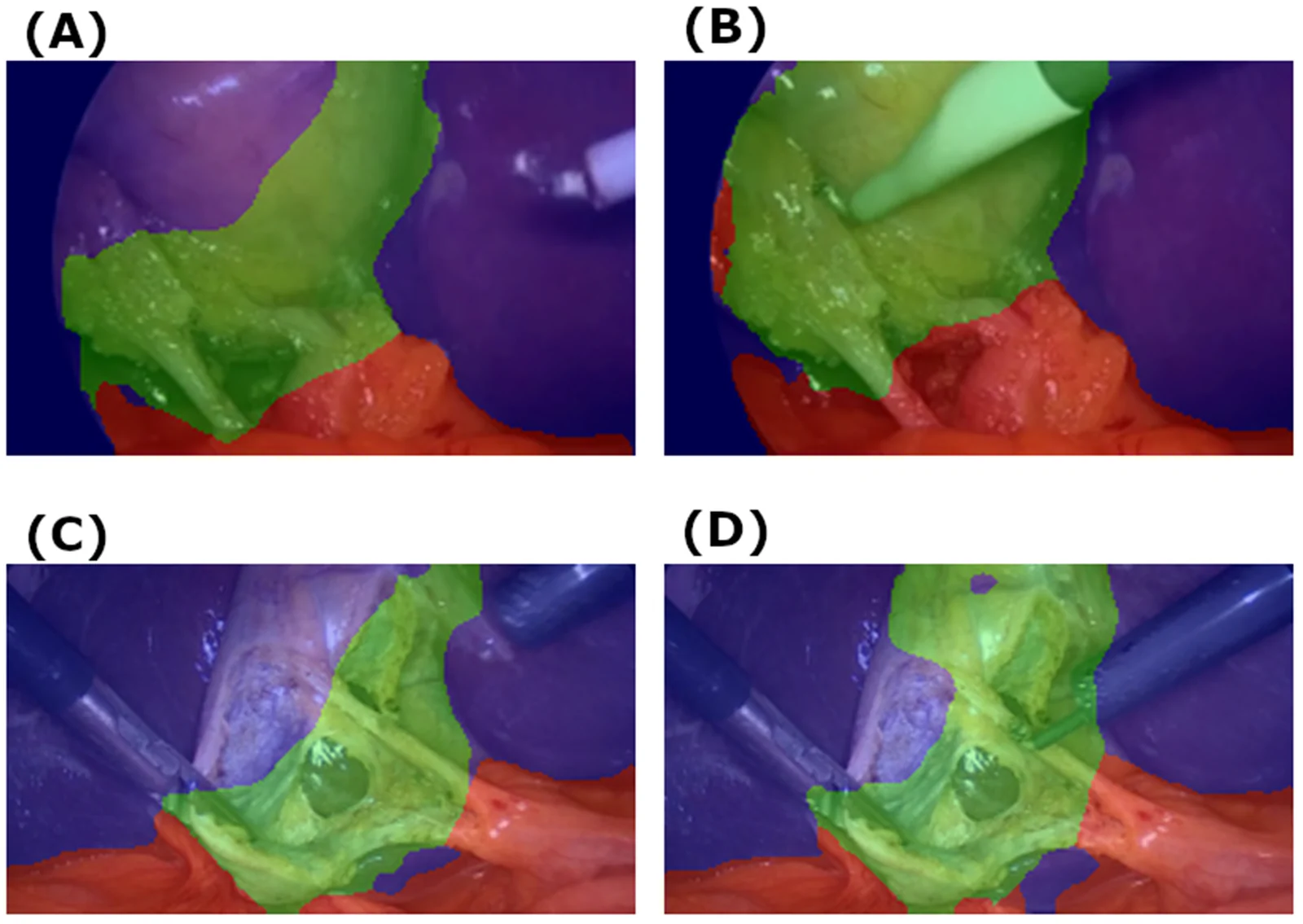
Prediction inconsistencies on two pairs of frames, where top row (A, B) is one pair, bottom row (C, D) is another pair, and frames within each pair are similar except for tool positions. Red highlights predicted dangerous zone, green – predicted safe zone, blue – predicted background. In both frames the presence of the surgical instrument has resulted in a larger predicted “safe” zone. Ground truth segmentations are not available for these frames.

### Baseline tool bias

For our best model trained without tool pasting or lighting augmentations, the per-video consistency scores (described in Section General principles of evaluation), averaged over all selected frame pairs within a video, range between 0.67 and 0.97, with a mean of 0.87 and standard deviation of 0.075, which shows that the model is sensitive to surgical tool position within the field of view. Examples of the prediction inconsistency on two pairs of frames, where frames within a pair are similar except for tool position, are presented in Fig 4.

### Tool bias mitigations

#### Manual tool tracking.

For each of the 23 short video segments and with manual tool tracking (described in the first part of Section Manual evaluation), for every selected pair of frames, we compared consistency of models trained with one and two iterations of tool pasting augmentation against the baseline model by subtracting the per-frame-pair consistency scores and averaging over the frame pairs in every video. Our findings aggregated over videos are presented in Fig 5. They show that for 18 out of 23 videos the model trained with one iteration of tool augmentations is more consistent than the baseline model, and that for 19 out of 23 videos the model trained with two iterations of tool augmentations is more consistent than the baseline model. Also, for 17 out of 23 videos two-iteration model is more consistent than one-iteration model.

**Fig 5:**
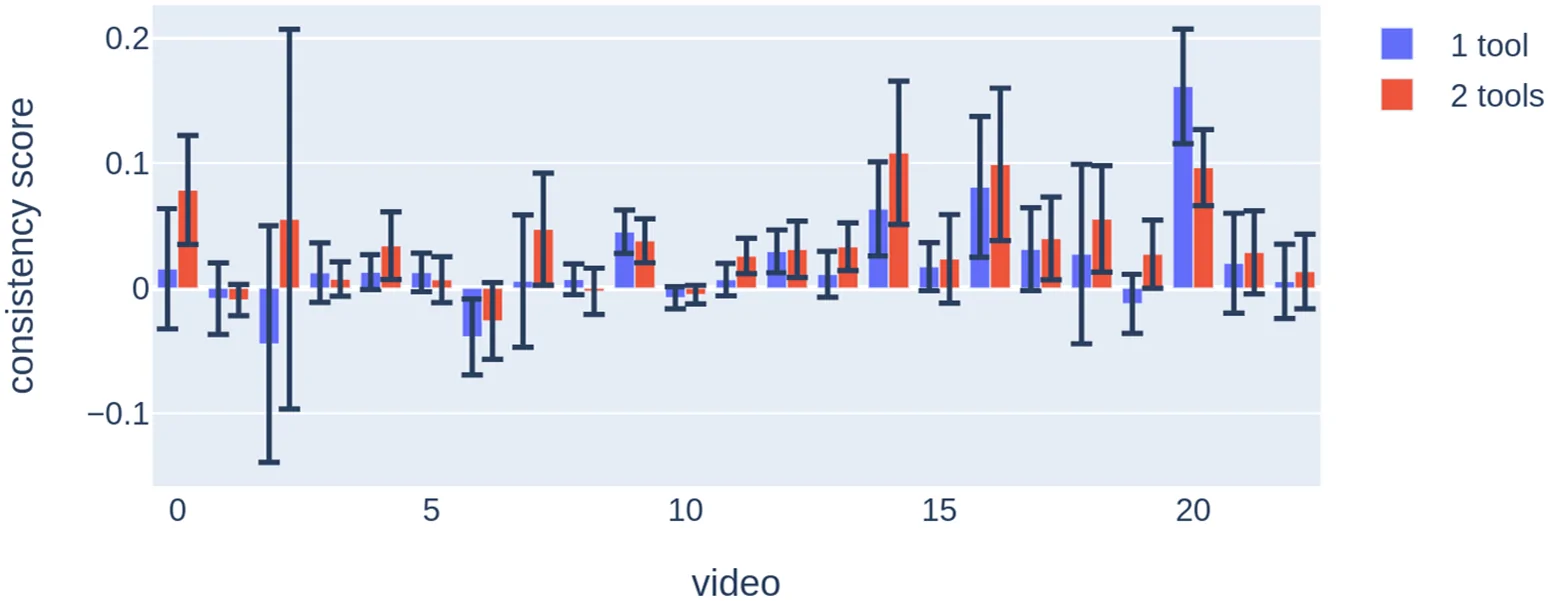
Per-video difference in consistency score of the models trained with one (blue) or two (red) iterations of tool pasting augmentation versus the baseline model, for every short video fragment. Error bars show standard deviation over 50 frame pairs.

To validate our claims statistically, we conducted three one-sided t-tests with paired samples, with the null hypotheses being “average per-video consistency scores for the model without any mitigations are not less than scores for the model with one iteration of tool pasting augmentation,” “average per-video consistency scores for the model without any mitigations are not less than scores for the model with two iterations of tool pasting augmentation,” “average per-video consistency scores for the model with one iteration of tool pasting augmentation are not less than scores for the model with two iterations of tool pasting augmentation,” with n = 23 for every test. The resulting p-values are, respectively, 0.016, $4.65\cdot 10^{-5}$ and 0.014, indicating significance and rejection of the null hypothesis in all three cases.

Our findings (summarized over all 23 videos) are presented in Table 1.

**Table 1:** A summary of consistency scores over all videos, for different tool bias mitigation strategies.

Mitigation strategy	Min	Max	Mean ± std
no augmentations	0.668	0.970	0.873 ± 0.075
paste 1 black shape	0.674	0.966	0.876 ± 0.073
paste 1 real tool image	0.698	0.971	0.893 ± 0.062
paste 2 black shapes	0.652	0.973	0.879 ± 0.084
paste 2 real tool images	0.765	0.965	0.908 ± 0.052
paste 3 real tool images	0.780	0.977	0.897 ± 0.048
DFR	0.667	0.945	0.872 ± 0.073
MARG-CTRL	0.714	0.961	0.897 ± 0.053

Applying tool pasting augmentation once or twice improves the model’s robustness, while merely covering parts of the image with tool-shaped black objects does not result in any improvement. No additional benefit is achieved from pasting three tools.

For our best model trained with a tool pasting augmentation applied once for every input image during training, the per-video consistency scores range between 0.7 (was 0.67 for the baseline model) and 0.97 (was 0.97), with a mean of 0.89 (was 0.87) and standard deviation of 0.062 (was 0.075). For our best model trained with a tool pasting augmentation applied twice for every input image, the per-video consistency scores further improved to range between 0.76 and 0.96, with a mean of 0.91 and standard deviation of 0.052. This demonstrates that applying tool pasting augmentation helps improve prediction consistency scores, and that applying the augmentation twice for every image amplifies the effect.

To study the effect increasing the amount of iterations of tool pasting augmentation has on tool bias, and to distinguish between the de-biasing effect from tool pasting and the regularization effect from obstructing parts of the image, we also conducted experiments with three iterations of tool pasting, as well as one or two iterations of pasting tools filled with black color. They show that making more than two iterations of tool pasting augmentation does not provide any further benefit, and that tool texture actually plays a significant role, as merely obstructing parts of the input image with tool-shaped black objects does not help reduce tool bias at all.

We also implemented two pre-existing shortcut learning mitigation methods: DFR [24] and MARG-CTRL [25] (marg-log and SD variants), with proper hyperparameter optimization. DFR did not provide any improvement in consistency, while MARG-CTRL still lagged behind the “two tools” version of our augmentation (Table 1). Another advantage of our approach is that MARG-CTRL’s derivation relies on the model being trained with cross entropy loss and is not necessarily compatible with, e.g., the Dice loss that is frequently used in segmentation settings, and also with object detection problem formulation. Our augmentation is data-driven and loss- and task-agnostic.

To statistically compare our method against MARG-CTRL, we took our strongest augmentation (“two tools” version), and fit a linear mixed-effects model to consistency scores calculated between pairs of video frames. For these purposes, we used lme4 library [34]. The mixed effect model included a fixed effect for the method of correction, and random effects for both the video and frame pair. Our method demonstrated significantly higher consistency scores than MARG-CTRL (p-value = 0.0148).

In Fig 6 we show the predictions of the model trained with two iterations of tool pasting augmentations on the same pairs of frames as in Fig 4. It can be seen that this model is much more consistent than the baseline model.

**Fig 6:**
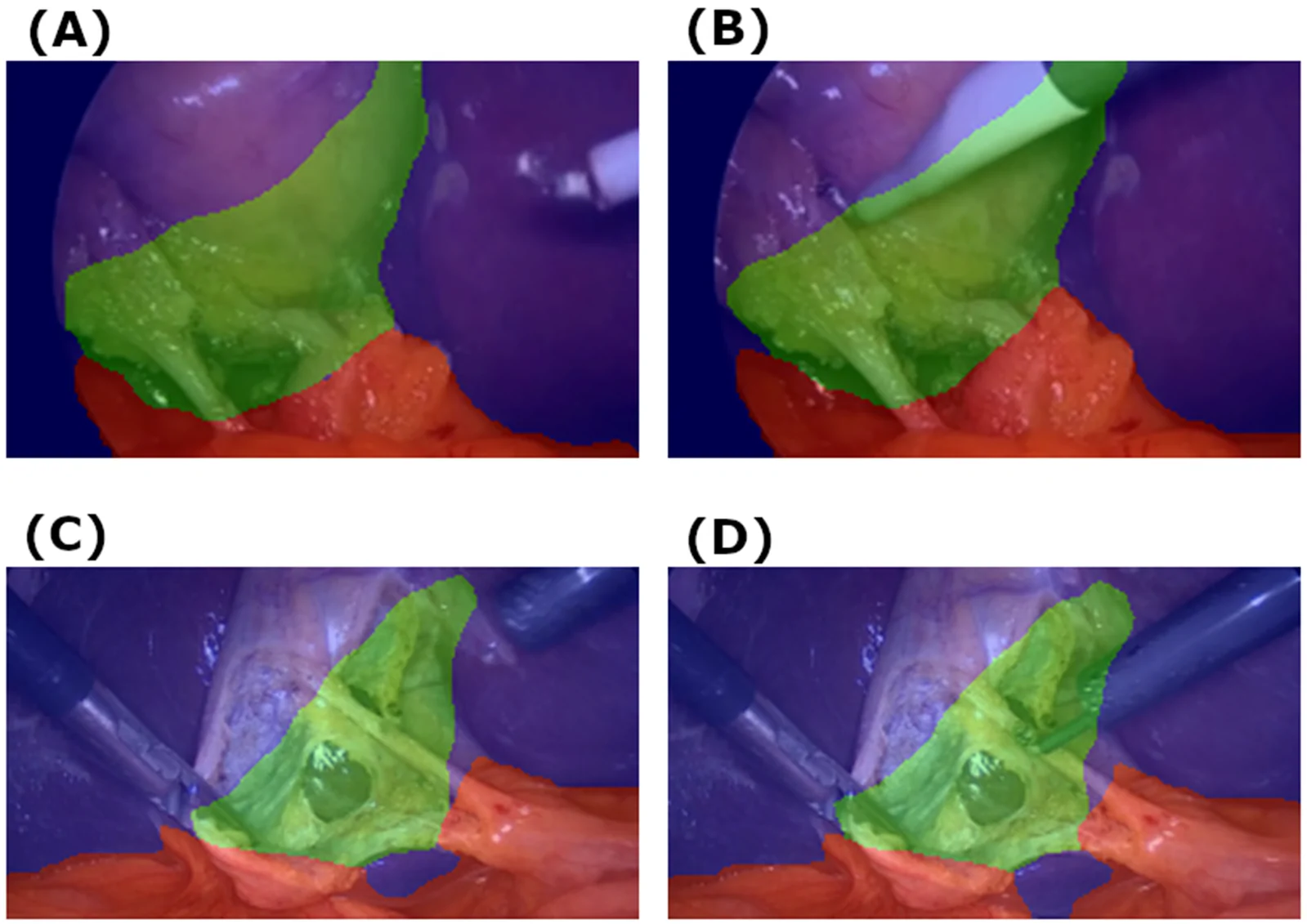
Predictions of the model trained with two iterations of tool augmentation on the same pairs of frames as were shown in Fig 4. Red highlights predicted dangerous zone, green – predicted safe zone, blue – predicted background. This model shows much better consistency than the baseline model (Fig 4). Ground truth segmentations are not available for these frames.

### Validation at scale

For the automated tool tracking on two videos from CholecTrack20 dataset [30] using the model trained on CholecInstanceSeg [29], Fig 7 shows by how many percentage points our best augmented model is more consistent than the baseline, as the tool movement between frames in a pair grows. This plot shows that the improvement in consistency is always positive and that it grows as the Hausdorff distance between tool positions grows. This is in agreement with our theory that larger discrepancies in tool locations should make it more challenging for the model to stay consistent, and that our mitigation strategies help ensure consistency.

**Fig 7:**
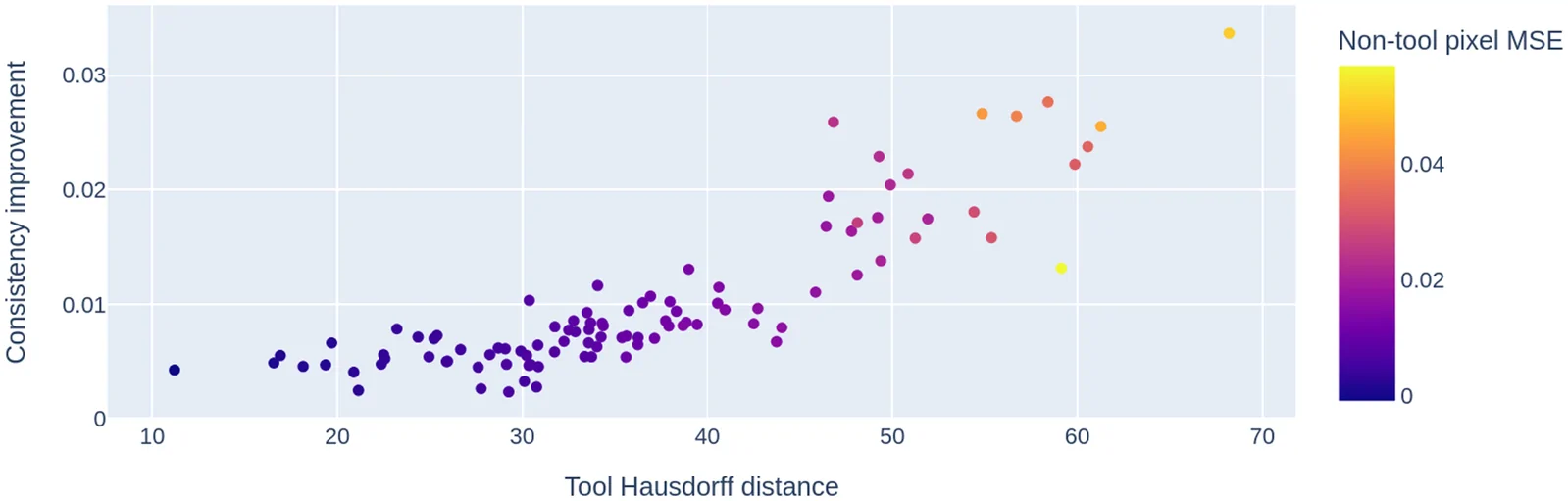
Safe/dangerous zone segmentation consistency improvement of tool-augmented model versus baseline, binned over non-tool pixel MSE and plotted against tool position Hausdorff distance.

Another takeaway from these experiments is that the surgical tool semantic segmentation model trained on CholecInstanceSeg dataset can be further used to extract more tool images and remove the need for manual tool annotation prior to using them for augmentation.

One could argue that numerical improvements of consistency may be misleading, because the model may be very consistent, but consistently wrong. In Section Segmentation performance we show that this is not the case for our augmented models, as they perform roughly on the same level as baseline models in terms of safe/dangerous zone segmentation metrics.

### Baseline lighting bias

As described in Section Evaluation, to evaluate lighting bias we assess how much the model’s predictions change when we consider predictions on the original input image to be “ground truth”, and compare predictions on images with local lighting adjustments against them. We found that the most drastic changes occur when the center of the simulated lighting blob is in the predicted safe zone, which is, importantly, the most surgically-relevant scenario, and further consider this case specifically. Fig 8A shows row-normalized confusion matrix (computed for every original image with all its transformations, then averaged over all original images), where every entry in position $c_{i},\;c_{j}$ shows what proportion of pixels that originally belonged to class $c_{i}$ belong to class $c_{j}$ in the transformed image. These results show that on average, 5% of the pixels originally predicted as a dangerous zone are classified as safe due to lighting adjustment. This is a dangerous mistake to make. Further, lighting adjustment causes another 5% of pixels originally predicted as belonging to dangerous zone to be predicted as background. Fig 8B shows fully-normalized confusion matrix (computed for every original image with all its transformations, then averaged over all original images), where every entry in position $c_{i},\;c_{j}$ shows what proportion of pixels originally belonged to class $c_{i}$ and belong to class $c_{j}$ in the transformed image. Fully-normalized confusion matrices are used to highlight zone size changes. Baseline model that does not use augmentation shows higher changes in zone sizes: dangerous zone shrinks by 7%, while safe zone and background grow by 2% and 1.5% respectively.

**Fig 8:**
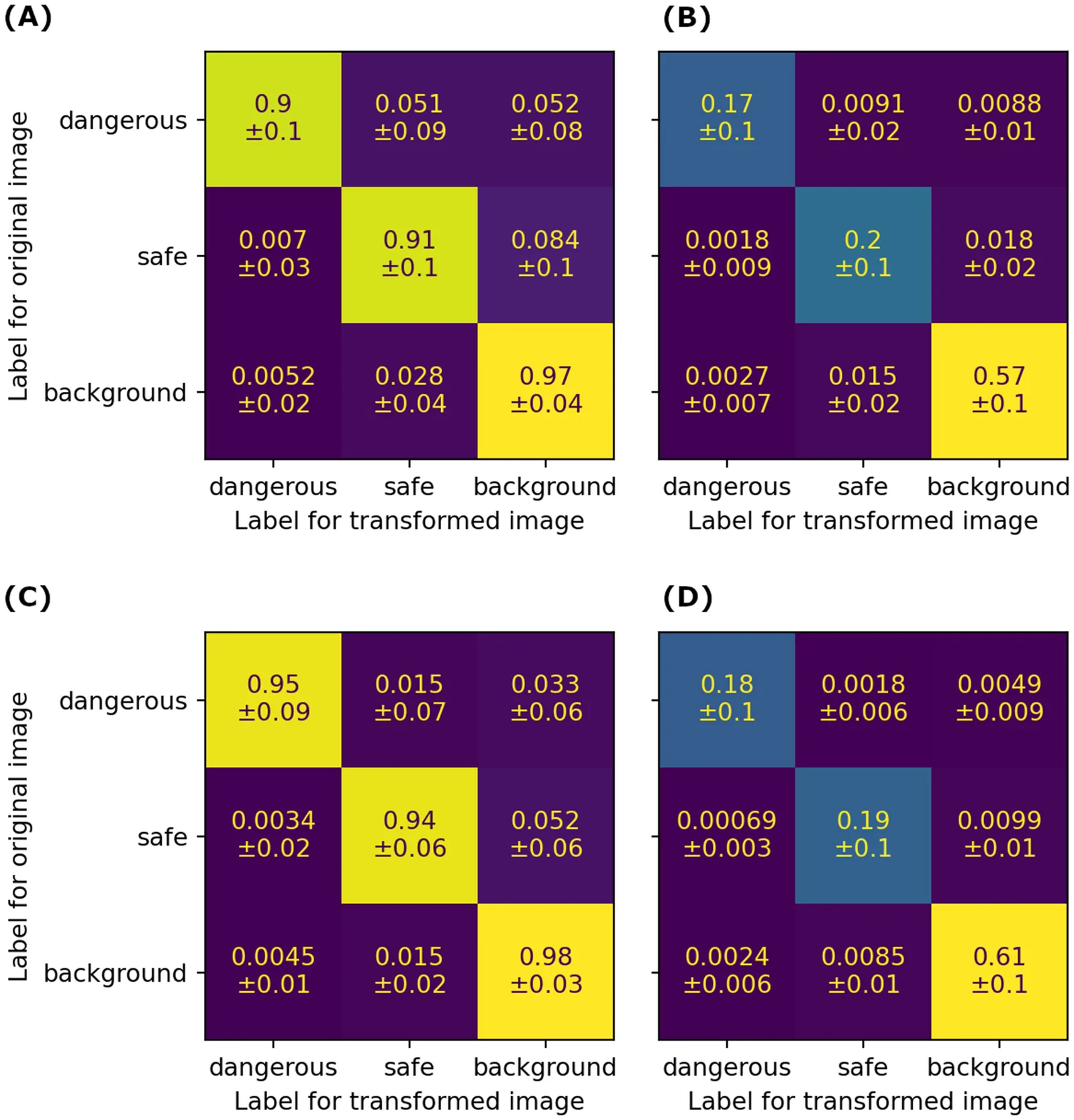
Confusion matrices for lighting bias. A: Row-normalized, without tool pasting or lighting augmentations. B: Fully-normalized, without tool pasting or lighting augmentations. C: Row-normalized, with lighting augmentations. D: Fully-normalized, with lighting augmentations.

### Lighting bias mitigations

Row-normalized and fully-normalized confusion matrices for the best model trained with random lighting augmentations are presented in Fig 8C and Fig 8D. They show that only 1.5% (was 5% without augmentations) of dangerous zone pixels can now be made to be predicted as safe zone, and 3.3% (was 5%) – as background. A model that uses lighting augmentation also results in much more consistent sizes of predicted zones (dangerous zone shrinks by 2%, safe zone shrinks by around 0.1% and background grows by less than 0.7%). Random lighting augmentation makes the model more robust to lighting changes, and helps prevent dangerous pixel misclassifications.

### Segmentation performance

While we have shown that models with lighting and tool augmentations are more consistent, it is also important to validate that their performance on the core task has not degraded as a result. Fig 9 shows row-normalized confusion matrices for segmentation quality for the baseline model and model with both two iterations of tool pasting augmentation and lighting augmentation. These confusion matrices show that the two models perform roughly on the same level in terms of segmentation quality, with the model with the augmentations performing slightly better, demonstrating that the proposed tool pasting and lighting augmentations do not affect segmentation quality on the dataset affected by both tool and location biases. One might wonder why the segmentation performance is almost unchanged given that the models trained with augmentations are supposed to generalize better by being more robust to spurious correlations. The reason is that such models are only supposed to generalize better to datasets not affected by these correlations, which, in this case, means real-life surgeries performed by less experienced surgeons, who may have difficulties with equipment positioning. Since the entirety of our dataset, including training, validation and testing splits, has such shortcuts, we expected the segmentation performance to remain roughly on the same level.

**Fig 9:**
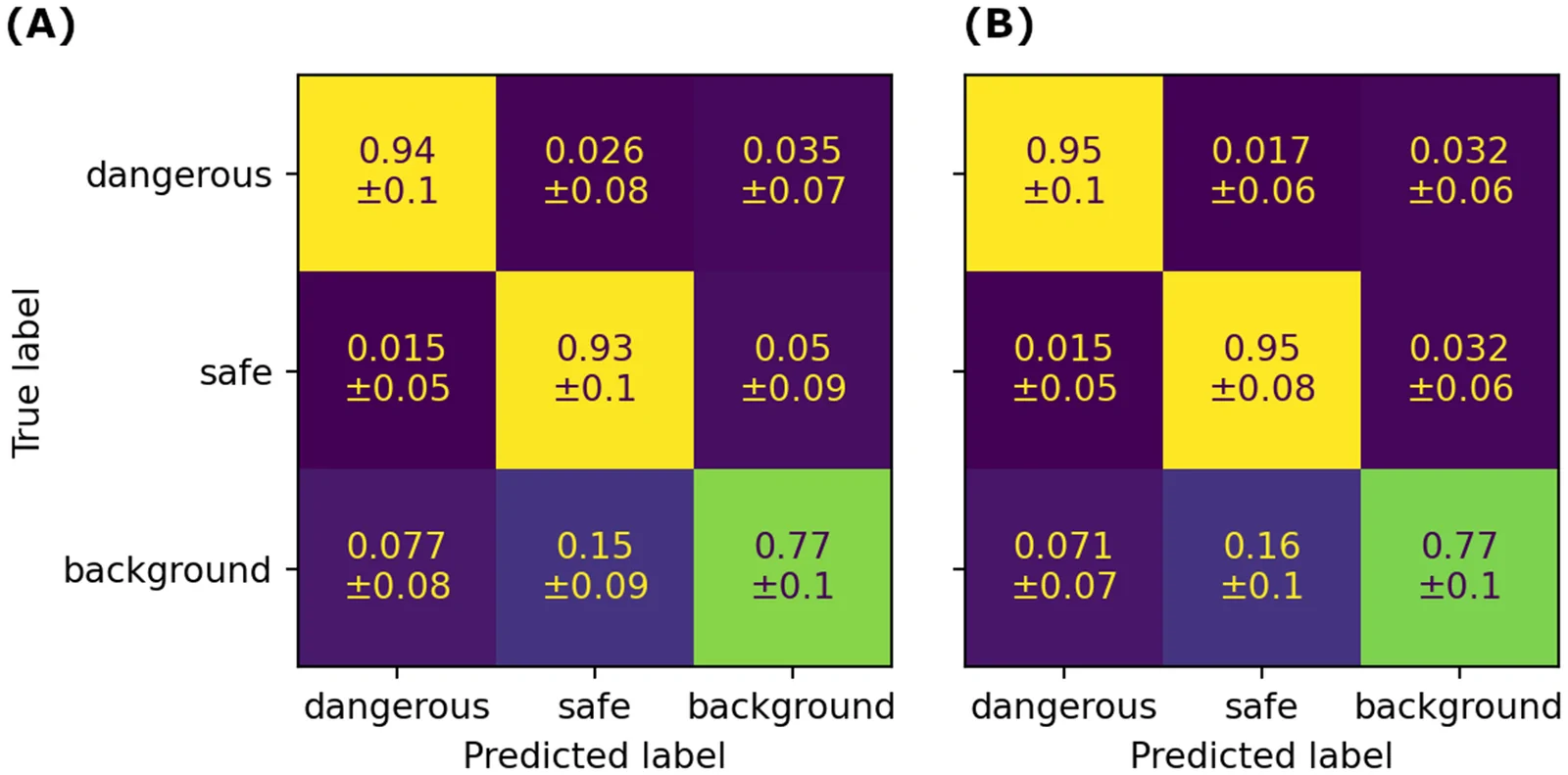
Row-normalized confusion matrices for segmentation quality. A: Baseline model. B: Model with both two iterations of tool pasting and lighting augmentations.

## Discussion

We have shown that surgical tool position and lighting direction act as confounders in training data for laparoscopic cholecystectomy computer vision models. Confounders of this kind elude standard machine learning evaluation: because the training, validation, and test splits all share the same shortcuts, a held-out test set, even one from a different institution, does not reveal the bias but rewards it. This is also why strong segmentation metrics do not necessarily reflect clinical utility. Dice, precision, and recall measure agreement with reference annotations on a same-distribution test set, so when a confounder pervades every split, high scores can coexist with behavior that is unreliable in the operating room.

Our consistency-based evaluation exposes this gap directly: a model can score well on static images yet change its safe and dangerous zone predictions when only an instrument’s position or the direction of lighting changes. Assessing clinical utility therefore requires measuring the stability of predictions under clinically realistic perturbations, not accuracy on a fixed test set alone.

These failure modes are clinically consequential. A predicted safe zone that expands to follow the instrument, or the baseline model’s reassignment of 5% of the pixels originally predicted as dangerous to the safe class when lighting is directed into the predicted safe zone (the most surgically relevant case, since it is where dissection occurs) are precisely the errors that could lead an operator to cut in a region that is in fact dangerous but that the system has labeled safe: the injury that safe and dangerous zone guidance exists to prevent. This risk is, moreover, unlikely to be evenly distributed. It would fall most heavily on surgeons in training and on those practicing in resource-limited settings, whose handling of instruments and lighting may depart from the expert patterns the model has implicitly encoded; a system that is least dependable for the operators who stand to benefit most from decision support inverts its own purpose and raises a question of equity alongside safety. We have not yet evaluated our models on data from less experienced operators, so this differential is a reasoned expectation grounded in the mechanism of the shortcut rather than a measured result; demonstrating it directly is an important direction for future work.

Our mitigation strategy addresses these confounders by decoupling anatomical recognition from equipment configuration, removing the model’s dependence on a cue that reflects who is operating rather than the anatomy itself. Because the augmentations are data-driven and agnostic to both the loss function and the model architecture, they can be incorporated into existing surgical AI training pipelines (and the consistency evaluation into existing validation protocols) without architectural change, lowering the barrier to adoption and likely generalizing beyond the SegFormer architecture used here. We expect both the problem and the mitigation to extend to other tasks on these datasets, such as identifying the liver, gallbladder, or hepatocystic triangle, or assessing whether the field of view and retraction are adequate, and to other surgical computer-vision problems. None of this comes at the cost of segmentation quality, which remains similar or slightly improved on our (necessarily biased) data (Section Segmentation performance).

As a limitation, we note that our augmented model failed to improve consistency under tool movements on 6 of 23 tested videos (see Fig 5). We conducted a manual analysis of these videos, and failed to identify a clear cause. One possible explanation is that during the manual evaluation phase the values of Hausdorff distance between tools and MSE between other pixels weren’t taken into account after they were used to select the frame pairs. In fact, these two measurements could be used to weigh the consistency scores of individual frame pairs during aggregation (as we did in the automated evaluation, summarized in Fig 7). We did not take into account these measurements during aggregation for manual evaluation, as that would complicate the analysis.

More broadly, the pervasiveness of these shortcuts in surgical datasets points to two needs: evaluation frameworks for medical computer vision that test consistency across variable conditions rather than accuracy on static images, and more diverse surgical datasets that deliberately capture variability in equipment positioning and other technical factors that would otherwise become hidden confounders. Together, these steps move the field toward surgical AI whose guidance can be trusted across the range of surgeons and operating conditions encountered in practice, a goal distinct from demonstrating strong performance on a single test dataset.

## Supporting information

S1 Fig Examples of tool images extracted from the video frames. (TIF)

S1 Table Overview of the datasets used in this study. (PDF)
