# Supplementary material for: Analysis and mitigation of equipment-induced shortcuts in AI models for laparoscopic cholecystectomy
Source: PLOS Digit Health. 2026 Oct 8;5(10):e0001421. doi: 10.1371/journal.pdig.0001421 (PMC13649130; doi:10.1371/journal.pdig.0001421)
Supplement: Supplementary file 1 [file pdig.0001421.s002.pdf]

S1 Table: Overview of the datasets used in this study.

Dataset / subset	Videos	Frames	Tool frames
**Dataset 1**			
Train	201	1,843	1,727
Validation	43	390	370
Test	45	394	376
Total	289	2,627	2,473
**Tool segmentation corpus**			
Train	33	20,546	18,464
Validation	11	6,730	6,073
Test	11	3,275	3,026
Total	55	30,551	27,563
**CholecTrack20**	2	106,738 @ 25 FPS	N/A
