# Supplementary material for: Analysis and mitigation of equipment-induced shortcuts in AI models for laparoscopic cholecystectomy
Source: PLOS Digit Health. 2026 Oct 8;5(10):e0001421. doi: 10.1371/journal.pdig.0001421 (PMC13649130; doi:10.1371/journal.pdig.0001421)
Supplement: Supplementary file 2 [file pdig.0001421.s003.pdf]

Responses to the reviewers

We thank the reviewers for their careful reading of our manuscript and positive feedback. We address their concerns below (see blue text). We have also uploaded a version of the manuscript with all changes tracked.

Reviewer #1

> *In this study, the authors identify and mitigate two shortcuts in the segmentation of laparoscopic cholecystectomy data with tools and lighting. The authors train a SegFormer model with data augmentation techniques, adding random tools or lighting to the images to mitigate the shortcuts. They show that training with such data augmentation improves the segmentation consistency across frames with different tool locations and lighting.*
>
> *Strengths:*
> *- The paper is overall clear and easy to follow. The figures and results nicely support the findings.*
> *- The study is complete, with both shortcut identification and mitigation.*
> *- The mitigation techniques based on data augmentation are simple and applicable to other models and other datasets.*
> *- The authors include both performance variability and statistical analysis.*

We appreciate the reviewer's recognition of our work's strengths, and thoughtful feedback, which we address as follows:

> *Weaknesses:*
> *- It would be nice to motivate or explain further the lack of ground truth in the tool bias evaluation. Performance is important since, as the authors mention, you may be more consistent but in a wrong way. Why not use frames with ground truth available to have an evaluation of performance as well? It could be because there is not enough in the data, but this is not really explained currently.*

We made sure to explain this in more detail in Section 4.3.2. The reason why safe/dangerous zone segmentation ground truth couldn't be used during tool bias evaluation is that this would require annotated frames that are temporally close enough to satisfy the "background is mostly static, only tools move" constraint. For each of the 289 surgical videos we have at most 10 annotated images that are spread uniformly along the video. Therefore, we use consistency of model predictions between similar

frames as a proxy.

We do, however, use available ground truth in Section 5.7 to demonstrate that the segmentation performance of the augmented model didn't degrade (and actually slightly improved) compared to the baseline, illustrating that the increase in consistency did not come at the cost of accuracy. We have clarified our explanation of all of these experiments to avoid confusion.

- There is no explanation of what the added tools are. Are they tools extracted from other images in the dataset or from online sources? It could also be relevant to discuss whether the choice of tools can impact the mitigation (e.g., if the tools in the mitigation and in the test set are different).

We made sure to elaborate on this in Section 4.3.3, and include a new Supplementary Figure 1 with images of the tools used for augmentations. The tools were extracted from the training subset of the video samples that underlie the dataset, including unlabeled frames that were never used for model training due to lacking the safe/dangerous zone segmentation ground truth. The tools include scalpels, graspers and cauterizers, and cover the same tool groups that can be seen in the validation and testing subsets. However, since training, validation and testing subsets of videos are disjoint, no leakage happens between them.

- The authors already cite [16] for their study on shortcut learning in medical segmentation, but I believe it should be mentioned that they also propose mitigating the shortcut through data augmentation/modification, which is related to the method proposed in this submission.

We made sure to make the citation more detailed in Section 3, and recognize that the augmentation techniques we propose are similar to those proposed in [16].

- Two comments on reproducibility:
- I may have missed it, but I don't see a link to the source code. It would be nice to have it publicly available.
- It would also be nice to include the final model's hyper parameters for the training.

We now make our code available at https://github.com/data-team-uhn/equipment_induced_shortcuts_in_lc and model weights available at https://zenodo.org/records/20746455. We include these links in the manuscript.

Reviewer #2

Protserov et al. present a well-designed study investigating strategies to mitigate shortcut learning related to tool position and lighting direction. The experimental design is robust, the analyses are comprehensive, and the topic is highly relevant to the development of trustworthy computer vision systems in healthcare. I believe the study is within the scope of PLOS Digital Health and addresses an important methodological challenge.
My main concern relates to the presentation of the work rather than the study itself. While the manuscript structure complies with PLOS Digital Health's publication requirements, I found it difficult to distinguish between methodological details, results, and interpretation. As a consequence, some important methodological information and discussion points were not always easy to locate. I believe the manuscript would benefit from clearer reporting in several areas outlined below.
The comments are ordered by importance, then by appearance in the article.

We appreciate the reviewer's recognition of our work's strengths, and thoughtful feedback, which we address as follows:

1. In the Abstract, the study's main focus and key takeaways could be communicated more clearly. The current abstract emphasizes the background of shortcut learning but provides comparatively limited detail on the study design, principal findings, and conclusions.

We have edited both the Abstract and the Introduction to include more details about our own study.

2. In Section 3.2 (Data), there should be a comprehensive overview of all datasets used in the study, including Dataset 1, CholecTrack20, and Endoscapes. A summary table that includes the number of videos, the number of frames, the image resolution, and the number of frames containing visible tools would be helpful. I also recommend reporting the characteristics of the augmented subsets alongside those of the original datasets, as augmentation is a key component of the study.

We made sure to provide thorough descriptions, including video counts, frame counts, utilized frame dimensionalities and purposes of all the datasets, both in prose in Section 4.2 and as Supplementary Table 1.

3. In Section 4.4 (Validation at Scale), important methodological details are introduced within the Results section. I recommend providing a sufficiently detailed methodological description in the Methods section. This would make it easier for

readers to understand the full picture of the study design and evaluate the reported findings.

Thank you for this catch. We have moved the methodological part of Validation at Scale to Methods (Section 4.3.4).

4. In Section 4.3 (Tool Mitigation Bias), the results indicate that the augmentation strategy does not improve consistency across all videos. This is an interesting observation that could benefit from further discussion.

We performed visual and automated analysis of video stability and motion characteristics on all 23 video fragments, but nothing in the videos stood out that would explain lower consistency when using the augmented model.

One possible explanation is that during the manual evaluation phase the values of Hausdorff distance between tools and MSE between other pixels weren't taken into account after they were used to select the frame pairs. In fact, these two measurements could be used to weigh the consistency scores of individual frame pairs during aggregation (as we did in the automated evaluation, summarized in Figure 8). We did not take these measurements into account during aggregation for manual evaluation, because that would complicate the analysis.

We now discuss this observation in the Discussion section of the manuscript.

5. In the Introduction, the authors briefly mentioned the evaluation of clinical utility as an important challenge. However, this topic receives limited attention in the remainder of the manuscript. I encourage the authors to expand the discussion of the clinical implications of their findings and how the proposed mitigation strategy may contribute to the development of clinically reliable surgical AI systems.

Thank you for the suggestion. We have edited the Discussion section to elaborate on this. Although our mitigations demonstrably improve prediction consistency, we cannot directly show that they improve generalization to settings in which these correlations are absent or weaker, such as surgeries performed by less experienced surgeons. Demonstrating that benefit would require evaluation on datasets that do not share these shortcuts. This highlights two broader needs: for evaluation frameworks in medical computer vision that test for consistency across variable conditions rather than accuracy on static images alone, and for more diverse surgical datasets that explicitly capture variability in equipment positioning and other technical factors that would otherwise become hidden confounders.

6. Please include additional information to improve reproducibility, including code, trained model weights, preprocessing pipelines, and the availability of other materials.

We now make our code available at https://github.com/data-team-uhn/equipment_induced_shortcuts_in_lc and model weights available at https://zenodo.org/records/20746455. We include these links in the manuscript.
