# Supplementary material for: Analysis and mitigation of equipment-induced shortcuts in AI models for laparoscopic cholecystectomy
Source: PLOS Digit Health. 2026 Oct 8;5(10):e0001421. doi: 10.1371/journal.pdig.0001421 (PMC13649130; doi:10.1371/journal.pdig.0001421)
Supplement: Peer review history

# Peer review: Decision Letter 0

**DOI:** 10.1371/journal.pdig.0001421.r001

**Authors:** Feng Liu, Adam Hulman

7 Jun 2026

Response to Reviewers Revised Manuscript with Track Changes Manuscript **Journal Requirements:**

1. Please amend your detailed Financial Disclosure statement. This is published with the article. It must therefore be completed in full sentences and contain the exact wording you wish to be published.

a. State what role the funders took in the study. If the funders had no role in your study, please state: “The funders had no role in study design, data collection and analysis, decision to publish, or preparation of the manuscript.”

b. If any authors received a salary from any of your funders, please state which authors and which funders.

2. We ask that a manuscript source file is provided at Revision. Please upload your manuscript file as a .doc, .docx, .rtf or .tex.

3. Please provide separate figure files in .tif or .eps format.

For more information about figure files please see our guidelines:  LINK

https://journals.plos.org/digitalhealth/s/figures

https://journals.plos.org/digitalhealth/s/figures#loc-file-requirements

4. Please provide an Author Summary. This should appear in your manuscript between the Abstract (if applicable) and the Introduction, and should be 150–200 words long. The aim should be to make your findings accessible to a wide audience that includes both scientists and non-scientists. Sample summaries can be found on our website under Submission Guidelines: [LINK]

https://journals.plos.org/digitalhealth/s/submission-guidelines#loc-parts-of-a-submission

5. Your manuscript is missing the following sections: Discussion. Please ensure these are present, and in the correct order, and that any references to subheadings in your main text are correct. An outline of the required sections can be consulted in our submission guidelines here: [LINK]

https://journals.plos.org/digitalhealth/s/submission-guidelines#loc-parts-of-a-submission

6. In the online submission form, you indicated that "All data used in this research is either already available and cited appropriately, or available upon request.".

All PLOS journals now require all data underlying the findings described in their manuscript to be freely available to other researchers, either

1. In a public repository,

2. Within the manuscript itself, or

3. Uploaded as supplementary information.

This policy applies to all data except where public deposition would breach compliance with the protocol approved by your research ethics board. If your data cannot be made publicly available for ethical or legal reasons (e.g., public availability would compromise patient privacy), please explain your reasons by return email and your exemption request will be escalated to the editor for approval. Your exemption request will be handled independently and will not hold up the peer review process, but will need to be resolved should your manuscript be accepted for publication. One of the Editorial team will then be in touch if there are any issues.

7. Some material included in your submission may be copyrighted. According to PLOS’s copyright policy, authors who use figures or other material (e.g., graphics, clipart, maps) from another author or copyright holder must demonstrate or obtain permission to publish this material under the Creative Commons Attribution 4.0 International (CC BY 4.0) License used by PLOS journals. Please closely review the details of PLOS’s copyright requirements here: PLOS Licenses and Copyright. If you need to request permissions from a copyright holder, you may use PLOS's Copyright Content Permission form.

Please respond directly to this email or email the journal office and provide any known details concerning your material's license terms and permissions required for reuse, even if you have not yet obtained copyright permissions or are unsure of your material's copyright compatibility.

Potential Copyright Issues:

Figures 1,2,3,4,6: Please confirm (a) that you are the photographer; or (b) provide written permission from the photographer to publish the photo(s) under our CC-BY 4.0 license.

**Additional Editor Comments (if provided):** **Reviewers' Comments:**

**Comments to the Author**

1. Does this manuscript meet PLOS Digital Health’s publication criteria?

Reviewer #1: Yes

Reviewer #2: Yes

**********

2. Has the statistical analysis been performed appropriately and rigorously?

Reviewer #1: Yes

Reviewer #2: Yes

**********

3. Have the authors made all data underlying the findings in their manuscript fully available (please refer to the Data Availability Statement at the start of the manuscript PDF file)?

The PLOS Data policy

Reviewer #1: Yes

Reviewer #2: No

**********

4. Is the manuscript presented in an intelligible fashion and written in standard English?

Reviewer #1: Yes

Reviewer #2: Yes

**********

Reviewer #1: In this study, the authors identify and mitigate two shortcuts in the segmentation of laparoscopic cholecystectomy data with tools and lighting. The authors train a SegFormer model with data augmentation techniques, adding random tools or lighting to the images to mitigate the shortcuts. They show that training with such data augmentation improves the segmentation consistency across frames with different tool locations and lighting.

Strengths:

- The paper is overall clear and easy to follow. The figures and results nicely support the findings.

- The study is complete, with both shortcut identification and mitigation.

- The mitigation techniques based on data augmentation are simple and applicable to other models and other datasets.

- The authors include both performance variability and statistical analysis.

Weaknesses:

- It would be nice to motivate or explain further the lack of ground truth in the tool bias evaluation. Performance is important since, as the authors mention, you may be more consistent but in a wrong way. Why not use frames with ground truth available to have an evaluation of performance as well? It could be because there is not enough in the data, but this is not really explained currently.

- There is no explanation of what the added tools are. Are they tools extracted from other images in the dataset or from online sources? It could also be relevant to discuss whether the choice of tools can impact the mitigation (e.g., if the tools in the mitigation and in the test set are different).

- The authors already cite [16] for their study on shortcut learning in medical segmentation, but I believe it should be mentioned that they also propose mitigating the shortcut through data augmentation/modification, which is related to the method proposed in this submission.

- Two comments on reproducibility:

- I may have missed it, but I don’t see a link to the source code. It would be nice to have it publicly available.

- It would also be nice to include the final model's hyper parameters for the training.

Reviewer #2: Review comments:

Protserov et al. present a well-designed study investigating strategies to mitigate shortcut learning related to tool position and lighting direction. The experimental design is robust, the analyses are comprehensive, and the topic is highly relevant to the development of trustworthy computer vision systems in healthcare. I believe the study is within the scope of PLOS Digital Health and addresses an important methodological challenge.

My main concern relates to the presentation of the work rather than the study itself. While the manuscript structure complies with PLOS Digital Health’s publication requirements, I found it difficult to distinguish between methodological details, results, and interpretation. As a consequence, some important methodological information and discussion points were not always easy to locate. I believe the manuscript would benefit from clearer reporting in several areas outlined below.

The comments are ordered by importance, then by appearance in the article.

1. In the Abstract, the study's main focus and key takeaways could be communicated more clearly. The current abstract emphasizes the background of shortcut learning but provides comparatively limited detail on the study design, principal findings, and conclusions.

2. In Section 3.2 (Data), there should be a comprehensive overview of all datasets used in the study, including Dataset 1, CholecTrack20, and Endoscapes. A summary table that includes the number of videos, the number of frames, the image resolution, and the number of frames containing visible tools would be helpful. I also recommend reporting the characteristics of the augmented subsets alongside those of the original datasets, as augmentation is a key component of the study.

3. In Section 4.4 (Validation at Scale), important methodological details are introduced within the Results section. I recommend providing a sufficiently detailed methodological description in the Methods section. This would make it easier for readers to understand the full picture of the study design and evaluate the reported findings.

4. In Section 4.3 (Tool Mitigation Bias), the results indicate that the augmentation strategy does not improve consistency across all videos. This is an interesting observation that could benefit from further discussion.

5. In the Introduction, the authors briefly mentioned the evaluation of clinical utility as an important challenge. However, this topic receives limited attention in the remainder of the manuscript. I encourage the authors to expand the discussion of the clinical implications of their findings and how the proposed mitigation strategy may contribute to the development of clinically reliable surgical AI systems.

6. Please include additional information to improve reproducibility, including code, trained model weights, preprocessing pipelines, and the availability of other materials.

**********

what does this mean?). If published, this will include your full peer review and any attached files.

**Do you want your identity to be public for this peer review?**  If you choose “no”, your identity will remain anonymous but your review may still be made public.

For information about this choice, including consent withdrawal, please see our Privacy Policy

Reviewer #1: No

Reviewer #2: No

**********

**Figure resubmission:**

**Reproducibility:**

To enhance the reproducibility of your results, we recommend that authors of applicable studies deposit laboratory protocols in protocols.io, where a protocol can be assigned its own identifier (DOI) such that it can be cited independently in the future. Additionally, PLOS ONE offers an option to publish peer-reviewed clinical study protocols. Read more information on sharing protocols at https://plos.org/protocols?utm_medium=editorial-email&utm_source=authorletters&utm_campaign=protocols

# Author comment: Author response to Decision Letter 1

**DOI:** 10.1371/journal.pdig.0001421.r002

30 Jun 2026

Attachment Submitted filename: responses_to_the_reviewers.pdf

# Peer review: Decision Letter 1

**DOI:** 10.1371/journal.pdig.0001421.r003

**Authors:** Feng Liu, Adam Hulman

10 Aug 2026

Analysis and Mitigation of Equipment-induced Shortcuts in AI Models for Laparoscopic Cholecystectomy

PDIG-D-26-00676R1

Dear Dr. Brudno,

We are pleased to inform you that your manuscript 'Analysis and Mitigation of Equipment-induced Shortcuts in AI Models for Laparoscopic Cholecystectomy' has been provisionally accepted for publication in PLOS Digital Health.

Before your manuscript can be formally accepted you will need to complete some formatting changes, which you will receive in a follow-up email from a member of our team.

Please note that your manuscript will not be scheduled for publication until you have made the required changes, so a swift response is appreciated.

IMPORTANT: The editorial review process is now complete. PLOS will only permit corrections to spelling, formatting or significant scientific errors from this point onwards. Requests for major changes, or any which affect the scientific understanding of your work, will cause delays to the publication date of your manuscript.

If your institution or institutions have a press office, please notify them about your upcoming paper to help maximize its impact. If they'll be preparing press materials, please inform our press team as soon as possible -- no later than 48 hours after receiving the formal acceptance. Your manuscript will remain under strict press embargo until 2 pm Eastern Time on the date of publication. For more information, please contact digitalhealth@plos.org.

Thank you again for supporting Open Access publishing; we are looking forward to publishing your work in PLOS Digital Health.

Best regards,

Adam Hulman, Ph.D.

Academic Editor

PLOS Digital Health

***********************************************************

**Reviewer Comments (if any, and for reference):**

Reviewer's Responses to Questions

**Comments to the Author**

Reviewer #1: All comments have been addressed

Reviewer #2: All comments have been addressed

**********

publication criteria?

Reviewer #1: Yes

Reviewer #2: Yes

**********

3. Has the statistical analysis been performed appropriately and rigorously?

Reviewer #1: Yes

Reviewer #2: Yes

**********

4. Have the authors made all data underlying the findings in their manuscript fully available (please refer to the Data Availability Statement at the start of the manuscript PDF file)?

The PLOS Data policy

Reviewer #1: Yes

Reviewer #2: Yes

**********

5. Is the manuscript presented in an intelligible fashion and written in standard English?

Reviewer #1: Yes

Reviewer #2: Yes

**********

Reviewer #1: I thank the authors for their answers and the revised article. All my comments have been addressed.

Reviewer #2: Thank you for your thoughtful revision. I appreciate the effort the authors put into addressing all of my comments. I have reviewed the revised manuscript and am satisfied that my concerns have been adequately addressed. I have no further comments and believe the manuscript is suitable for publication.

**********

what does this mean?). If published, this will include your full peer review and any attached files.

**Do you want your identity to be public for this peer review?**  If you choose “no”, your identity will remain anonymous but your review may still be made public.

For information about this choice, including consent withdrawal, please see our Privacy Policy

Reviewer #1: No

Reviewer #2: No

**********
